# Functional Connectivity among Spikes in Low Dimensional Space during Working Memory Task in Rat

**DOI:** 10.1371/journal.pone.0091481

**Published:** 2014-03-21

**Authors:** Mei Ouyang, Shuangyan Li, Xin Tian

**Affiliations:** Laboratory of Neurobiology in Medicine, School of Biomedical Engineering, Tianjin Medical University, Tianjin, China; Tel Aviv University, Israel

## Abstract

Working memory (WM) is critically important in cognitive tasks. The functional connectivity has been a powerful tool for understanding the mechanism underlying the information processing during WM tasks. The aim of this study is to investigate how to effectively characterize the dynamic variations of the functional connectivity in low dimensional space among the principal components (PCs) which were extracted from the instantaneous firing rate series. Spikes were obtained from medial prefrontal cortex (mPFC) of rats with implanted microelectrode array and then transformed into continuous series via instantaneous firing rate method. Granger causality method is proposed to study the functional connectivity. Then three scalar metrics were applied to identify the changes of the reduced dimensionality functional network during working memory tasks: functional connectivity (GC), global efficiency (E) and casual density (CD). As a comparison, GC, E and CD were also calculated to describe the functional connectivity in the original space. The results showed that these network characteristics dynamically changed during the correct WM tasks. The measure values increased to maximum, and then decreased both in the original and in the reduced dimensionality. Besides, the feature values of the reduced dimensionality were significantly higher during the WM tasks than they were in the original space. These findings suggested that functional connectivity among the spikes varied dynamically during the WM tasks and could be described effectively in the low dimensional space.

## Introduction

Working memory (WM) refers to a brain system that provides temporary storage and manipulation of the information necessary for complex cognitive tasks [1]. The prefrontal cortex (PFC) is thought to play a critical role in memory organization and executive functions during working memory tasks [2], [3]. A prevailing view is that WM is mediated by the activities of medial prefrontal cortex (mPFC) neurons [4]–[6]. Up till now, the neural mechanism of WM is still an open question.

The functional connectivity has been previously investigated to explore the mechanism of working memory [7], and provides novel insights into psychiatric and neurological disorders [8], [9]. For example, abnormalities of the brain functional connectivity network have been observed in Alzheimer's disease [10]–[12], which is thought to relate to the declination of WM. In recent years, there have been a numerous studies on functional connectivity among neuronal signals at different levels and scales [13]. A powerful technique to extract such connectivity from data is Granger causality connectivity analysis (GCCA). GCCA, originating from the field of economics and being widely used in neuroscience [14], is an effective method to investigate the interactions between variables [15].

Recently, GCCA has been widely used to extract functional connectivity from macroscopic neuronal signals such as fMRI [16], EEG and MEG [17], [18]. Meanwhile, identifying functional connectivity between the microscopic neuronal signals, such as spikes, is also very important to understand how the brain orchestrates information processing at the single-cell and population levels [19]. Besides, these microscopic neuronal signals recorded by micro-electrode array, have good temporal resolution.

Principal component analysis (PCA) is a dimensionality reduction technique to extract the important information by maximally concentrating the energy contained in the signals in a smaller number of components. Following high-dimensionality reduction by PCA, functional connectivity of fMRI was identified and visualized [20] to reveal novel insights into dynamic brain connectivity [21]. Hu et al. performed PCA on spikes recorded in rats' motor cortices through brain-machine interfaces when rats were involved in real-time control tasks [22]. Meanwhile, investigation has suggested that the behavior onset of a skilled reaching task could be predicted using a smaller number of principal components extracted from the population activity [23]. Moreover, Zhou et al. have proposed an approach to investigate functional connectivity by combining PCA and GCCA [24].

Analyses of functional connectivity might serve as an effective description of the neural mechanism underlying information processing in small scale of brain (i.e. mPFC) during the WM tasks. In this study, we investigated the functional connectivity variation among spikes during the WM tasks. Then, we investigate the functional connectivity by combining PCA and GCCA to explore whether these variations of the network can be effectively described in low dimensional space.

## Materials and Methods

### Experiment and Data acquisition

This study was approved by the Institutional Animal Care and Use Committee of Tianjin Medical University under the NIH Guide for the Care and Use of Laboratory Animals.

**Figure 1:**
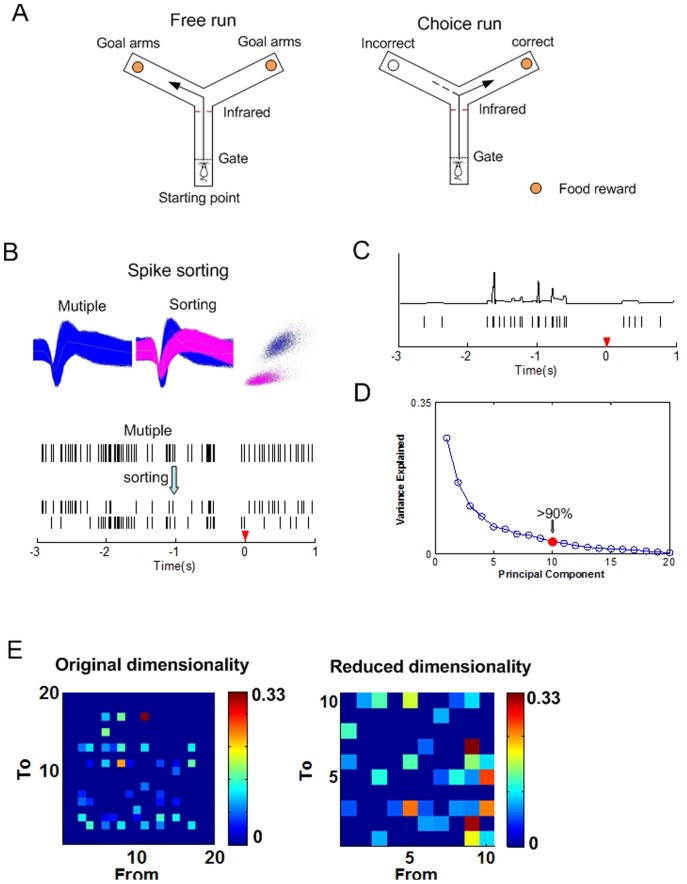
The Y maze working memory task and preprocessing of the data. (A) The Y maze working memory task. For the Y maze, dashed lines represent the removable guillotine doors. The occurrence of behavioral event is detected by an infrared sensor. Food rewards are located at the ends of goal arms. Arrow shows in the right plot possible correct path, dotted line in the right plot shows possible incorrect path. (B) Processes of spike sorting. (C) Rastergram of the spike trains recorded during the Y-maze task (3 s pre and 1 s post the tripping time) and the converted continuous series. The red triangle denotes the tripping time of the ‘choice run’ behavioral event in the Y-maze. (D) Plot of the principal components obtained from the continuous time series. The first 10 principal components (PCs) account for over 90% energy of the total variables. (E) Granger causality matrices in the original (left) and in the reduced dimensionality (right).

Male Sprague-Dawley rats weighing 300–350 g were given a two-day food limitation for 2 h per day to maintain body weight of no less than 85% of normal weight. Then, the rats were adapted to the Y-maze (Fig. 1A) for another two days. After habituation, rats were given training sessions on a Y-maze working memory task per day until the correct rate of the performance was over 85%. The Y maze working memory task (Fig. 1A) was simply described here: Each trial of the task included a free choice run and a delayed alternation run. In the free choice, the rat could get a small piece of food reward when it arrived at either end B or end C. After consuming the reward, the rat returned to the start A and waited for 5 seconds to make a ‘choice run’. During this phase, the rat could only get the reward at the end of the arm which it had not entered previously. After a trial, the animal went back to A to start the next trial.

After reaching the performance criterion, the rats were anesthetized with chloral hydrate (350 mg/kg) under aseptic conditions. Meanwhile, 2×8 nickel-cadmium microelectrode array (impedance less than 1 MΩ) were implanted into the rats mPFC according to the rat brain stereotaxic coordinates [AP: 2.5–4.5 mm, ML: 0.2–1.0 mm, DV: 2.5–3.5 mm].

After recovery, multi-channel neural signals were recorded in rat mPFC through a Cerebus Acquisition System (Cyberkinetics, USA) during the WM tasks on the Y-maze. Then, spikes (high pass filter: 250–7500 Hz, sampled at 30 kHz) exceeding the voltage threshold were stored with the time stamps per channel. After that, spike-sorting was performed to classify different neuronal firings by offline sorter (Fig. 1B, Plexon). The occurrence of behavioral events was marked by an infrared sensor in the Y-maze and was defined as the ‘tripping point’ [25].

### Conversion of spikes into continuous series

As spikes are series of discrete action potentials of neurons and consist of point processes, they cast a challenge on multivariate autoregressive (MVAR) model of GCCA. In that case, spikes should be firstly converted into continuous time waveforms. Considering that both rate and temporal information might be important for assessing the interaction among neurons, we here used a method which was applicable for dealing with short, sparse spikes trains to generate a continuous time series more suitable for MVAR modeling [26]. The basic idea of this method was to convert the spikes into continuous temporal series of instantaneous firing rate (IFR). Briefly, the procedure included three steps:

Firstly, the IFRs were obtained by calculating the inverse of the interspike intervals.
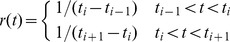
(1)where *t_i-1_*, *t_i_* and *t_i+1_* denoted three successive spike occurring times.

Then a small time interval δT (much less than the average interspike interval T) was chosen to yield the instantaneous integrated rate (*f(t)*).
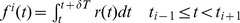
(2)


Finally, a continuous-time rate series was obtained by smoothing out the functions in time by spline interpolation methods (Fig. 1C, upper).

### Granger causality

The measure of Granger causality are based on the notion that a variable x_1_ can be said to cause another variable x_2_ if the information in the past of x_1_ can help forecast the future of x_2_ with better accuracy [27], [28].

In this study, the number of the variables was larger than two. In that case, conditional G-causality was applied to measure the causal influences between the pair of signals in the multivariate data set X.
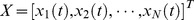
(3)


To illustrate conditional G-causality, suppose that the temporal signals (X) can be represented by a multivariate autoregressive (MVAR) model.
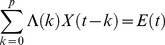
(4)In which E(t) is a vector of multivariate zero-mean uncorrelated white noise process. 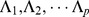 are N by N matrices of model coefficients while p is the model order chosen with the Bayesian information criteria (BIC) for MVAR process. Then, the conditional Granger causality was calculated.

In the time domain, the Granger causality from x_2_(t) to x_1_(t) conditional on the other signals is defined as:
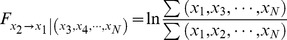
(5)where 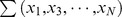
is the variance of the noise in the regression of the variables data set without the x_2_, and 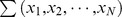 is the variance in the regression of all variables of X, both variances being associated with x_1_ variable. Then, the Bonferroni correction was applied to assess the statistical significance of the G-causality interaction (p = p_nom_/n(n-1), p_nom_ = 0.01) [15]. Interactions which do not reach statistical significance are set to zero. In addition, the path lengths were determined by taking the inverse of the strength of the granger causality connection. Then, graph theoretical measures were applied to estimate the connectivity as it provided an effective and informative way to explore the network properties [29].

### Feature calculations

Focusing on the effectiveness of the network information transmission, the global efficiency (E) and the causal density (CD) were selected to explore the variance of network properties during the WM tasks in this study.

The global efficiency is defined as the inverse of the harmonic mean of the minimum path length between each pair of the elements [30].
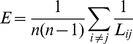
(6)where L_ij_ is the mean of the minimum path length between nodes *i* and *j* (neurons or principle components). E = 0 indicates that there is no path between neurons or principle components (PCs). High value of E reveals strong effect of the parallel information transfer among nodes [31].

The causal density of the data set X is a global measure of causal interactivity. It is defined as the mean of all pairwise causalities between the elements, conditioned on the remainder of the system.
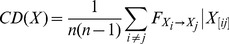
(7)where 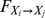 is the value of the Granger causality strength from node i to node j and X_[i,j]_ denotes the subsystem of X without X_i_ and X_j_. When all the statistically significant interactions are set to 1, the value of CD will be bounded into the range of [0, 1]. High value of the CD indicates more causal links observed in the network and is obtained only when the connectivity among the elements is globally coordinated.

### Connectivity among principal components

PCA is a statistical analysis technique that can be used for feature extraction by seeking the linear combinations of the original variables to derive new variables which capture the maximal variance. Given a matrix with *n* variables and *m* observations for each variable, the goal of PCA is to reduce the dimensionality of the inputs by projecting them onto the reduced *r* directions. These *r* new variables are mutually uncorrelated and orthogonal. They, together, account for as much of the energy contained in the original n variables as possible [32]. Investigations have demonstrated that using PCA may improve upon application of existing Granger causality method in the study of brain effective connectivity [33]. In this study, we selected *r* new variables (principal components) accounted for over 90% energy of the original variables. As an example shown in Fig. 1D, the first 10 principal components (PCs) accounted for over 90% energy of the total variables. In that case, thirteen was chosen as the number of the PCs. These low dimension principal components from the continuous time series of spikes were further analyzed to investigate whether they could facilitate the subsequent Granger causality analysis (Fig. 1E, right).

## Results

In order to investigate dynamic variations of the functional connectivity during the WM tasks in rat mPFC, the average values of granger causality matrix obtained from the continuous spikes (GC) were calculated (80 trials of 6 rats). The network was identified by effective connections among the continuous spikes. Meanwhile, we analyzed the global efficiency (E) and the causal density (CD) in order to feature the changing tendencies of the parallel information transfer and the global coordination of the network based on granger causality matrix.

Furthermore, PCA was applied to the continuous spikes to extract the principle components (PCs). As a comparison, the average values of granger causality matrix obtained from the PCs (GC_PC_) were also analyzed. In addition, global efficiency and causal density of the network identified by functional connections among the PCs, denoted by E_PC_ and CD_PC_, were subsequently calculated and compared to explore the effectiveness of describing the functional connectivity by using PCA.

### Dynamic variations of functional connectivity during the WM tasks

The changing tendencies of the functional connectivity were analyzed during the correctly performed WM tasks (4 s pre and 2 s post the tripping point). Accordingly, the period of the WM task was divided into six 1 s length bins which were defined as A_0_, A_1_, A_2_, A_3_, A_4_ and A_5_ from the beginning to the end. A_0_ was defined as the working memory beginning state (WMBS).

**Figure 2:**
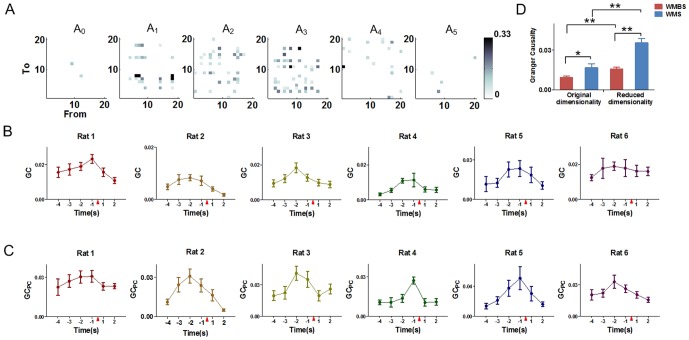
Dynamic variations of granger causality during working memory tasks. The data are divided into six 1(4 s pre and 2 s post the tripping time). The red triangle indicates the tripping time of the infrared sensor in the Y-maze. (A) Dynamic variations of the granger causality matrixes during a working memory task of rat 1. (B) Variations of the GC values in the original dimensionality during the working memory tasks of each rat (mean±SEM). (C) Variations of the GC_PC_ values in the reduced dimensionality during the working memory tasks of each rat (mean±SEM). (D) Comparisons of granger causality (mean±SEM). The granger causality values of the original and the reduced dimensionality are both significantly higher at the working memory state (WMS) than at the beginning state (WMBS). Besides, the granger causality levels in the original dimensionality at the WMS and the WMBS are significantly lower than those in the reduced dimensionality (80 trials for 6 rat, paired sample t-test, ^*^ P<0.05, ^**^ P<0.01).

Granger causality analysis was subsequently applied to investigate the changing tendencies of the functional connectivity. As an example shown in Fig. 2A, the number of the effective connections whose values were statistically significant became larger during the WM task (at A_2_ and A_3_). Then the effective connections decreased to smaller numbers after the tripping point (at A_4_ and A_5_).

The changing tendencies of the GC levels were subsequently analyzed. Results showed that the GC levels among the continuous spikes were dynamically varied from A_0_ to A_5_ during the WM tasks (Fig. 2B). The values of the GC increased to the maximum (defined as the working memory state (WMS)) from the beginning, and then decreased in all six rats. The maximum values of GC appeared 2 s pre (A_2_, for Rat 2, Rat 3 and Rat 6) or 1 s pre (A_3_, for Rat 1, Rat 4 and Rat 5) the tripping point. Of note, the GC values of the WMS were significantly higher than those of the WMBS (Fig. 2D, 80 trials of 6 rats, original dimensionality: ^*^ P<0.05). However, further comparisons between the WMBS and the WMS for each rat respectively suggested statistical differences of the GC values only in 4 rats (Table 1, paired sample t-test, ^*^ P<0.05, ^**^ P<0.01).

**Table 1:** Comparisons of the granger causality of the original and the reduced dimensionality at WMBS and WMS^a^.

	N^b^	Original dimensionality		Reduced dimensionality	
		WMBS	WMS	WMBS	WMS
Rat 1	26	0.0156±0.0030	0.0233±0.0026**	0.0225±0.0062	0.0308±0.0046*/▵
Rat 2	20	0.0048±0.0011	0.0083±0.0012*	0.0112±0.0022▵▵	0.0310±0.0053**/▵▵
Rat 3	28	0.0093±0.0019	0.0183±0.0029*	0.0196±0.0046▵▵	0.0410±0.0067**/▵▵
Rat 4	36	0.0054±0.0012	0.0113±0.0038	0.0105±.00185▵▵	0.0271±0.0027**/▵▵
Rat 5	28	0.0115±0.0059	0.0235±0.0162	0.0134±0.0038	0.0507±0.0155**/▵
Rat 6	22	0.0125±0.0017	0.0189±0.0024*	0.0203±0.0051	0.0329±0.0062*/▵

a The values are the mean ± SEM of the average granger causality values of each rats (paired sample *t* test).

b Total number of the compared groups.

* p<0.05 compared to the granger causality values at the WMBS in the original dimensionality or the reduced dimensionality.

** p<0.01 compared to the granger causality values at the WMBS in the original dimensionality or the reduced dimensionality.

▵ p<0.05 compared to the granger causality values of the original dimensionality at the WMBS or at the WMS.

▵▵ p<0.01 compared to the granger causality values of the original dimensionality at the WMBS or at the WMS.

Subsequently, the PCA was applied on the continuous spikes to extract the PCs to construct the reduced dimensionality network. Investigations of the GC_PC_ showed that the values of GC_PC_ presented similar increasing tendencies during the WM tasks and decreased after reaching the largest values (Fig. 2C). Of note, the occurring time of the GC_PC_ highest levels were the same as the GC. Difference between the GC_PC_ levels of the WMBS and the WMS was statistically significant (Fig. 2D, 80 trials of six rats, reduced dimensionality: P<0.01). Most importantly, further comparisons between the GC_PC_ of different state for each rat presented statistical differences for all 6 rats (Table 1, paired sample t-test, ^*^ P<0.05, ^**^ P<0.01).

To further investigate the effectiveness of using PCA, we then compared the values of GC and GC_PC_ at both the WMBS and the WMS. Results showed significant differences between GC and GC_PC_ values at both the WMBS and the WMS (Fig. 2D, paired sample t-test, P<0.01). Of note, comparisons between the GC_PC_ and GC at WMBS showed significant difference only in 3 rats (Table 1, paired sample t-test, ^▵^ P<0.05, ^▵▵^ P<0.01), though the values of GC_PC_ were higher than the GC in all the rats.

Thus, the values of the GC and the GC_PC_ were both significantly increased during the WM tasks. Besides, the variations of the functional connectivity could be better described in the reduced dimensionality.

### Dynamic variations of the features during WM

As mentioned in section 2, the causal density and the global efficiency were selected to feature the functional connectivity network. E, CD of the network constructed from the original continuous spike series and E_PC_, CD_PC_ of the network constructed from the PCs were calculated during the correct WM tasks to describe the variations of the functional connectivity.

**Figure 3:**
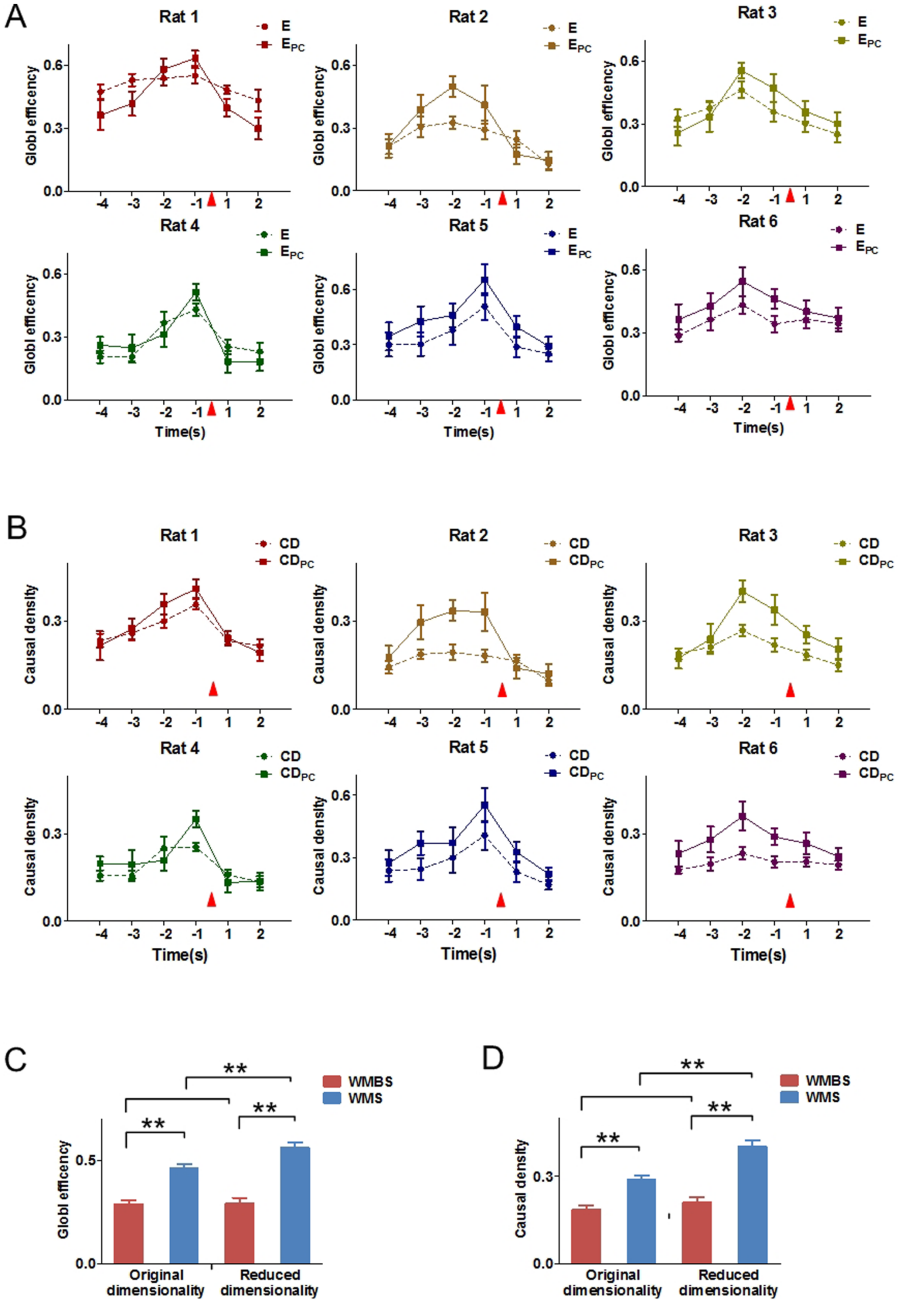
Dynamic variations of global efficiency and causal density both in the original and in the reduced dimensionality during working memory tasks, 6 rats respectively. (A) The dynamic variations of global efficiency in the original dimensionality (dashed lines) and the reduced dimensionality (solid lines) during the WM tasks, 6 rats respectively (mean±SEM). (B) The dynamic variations of causal density in the original dimensionality (dashed lines) and the reduced dimensionality (solid lines) during WM tasks, 6 rats respectively (mean±SEM). The red triangle indicates the tripping time of the infrared sensor in the Y-maze. (C) Comparisons of global efficiency (mean±SEM). The values of the global efficiency in both the original and the reduced dimensionality are significantly higher at the WMS than at the WMBS. The E values in the original dimensionality at the WMS are significantly lower than the E_PC_ values in the reduced dimensionality (80 trials for 6 rat, paired sample t-test, ^**^ P<0.01). No significant difference is found at the WMBS (80 trials for 6 rat, paired sample t-test, P>0.05). (D) Comparisons of causal density (mean±SEM). The values of the causal density in both the original and the reduced dimensionality are significantly higher at the WMS than at the WMBS. The CD values in the original dimensionality at the WMS are significantly lower than the CD_PC_ values in the reduced dimensionality (80 trials for 6 rat, paired sample t-test, ^**^ P<0.01). No significant difference is found at the WMBS (80 trials for 6 rat, paired sample t-test, P>0.05).

The variation trends of E, CD, E_PC_ and CD_PC_ were shown in Fig. 3. They all increased during the WM tasks and decreased after reaching the maximum values (Fig. 3A and B). Besides, the maximum values of all the features (E, CD, E_PC_ and CD_PC_) occurred at A_2_ (Rat 2, Rat 3 and Rat 6) and A_3_ (Rat 1, Rat 4 and Rat 5). Of note, the highest feature levels appeared at the same time with GC and GC_PC_. In other words, they presented similar changing tendencies during the WM tasks.

To investigate whether there were statistical differences between the features values of the WMBS and WMS, we further compared the average values of the features values of these two states for each rats respectively. As shown in Table 2 and Table 3, compared with the values of the WMBS, the feature values were higher at the WMS. Besides, statistical differences were found between the features of WMBS and WMS in the reduced dimensionality for all the rats (E_PC_ and CD_PC_, Table 2 and Table 3, paired sample t-test, ^**^ P<0.01, ^*^ P<0.05). For the networks constructed from the continuous spikes, however, significant differences between the two states (WMBS and WMS) could not be found for 5 rats (Table 2 and Table 3, paired sample t-test, ^**^P<0.01, ^*^P<0.05).

**Table 2:** Comparisons of the global efficiency of original and reduced dimensionality at WMBS and WMS^a^.

	N^b^	Original dimensionality		Reduced dimensionality	
		WMBS	WMS	WMBS	WMS
Rat 1	26	0.47±0.04	0.55±0.04	0.36±0.07	0.63±0.04**/▵
Rat 2	20	0.21±0.04	0.33±0.03*	0.22±0.06	0.50±0.05**/▵▵
Rat 3	28	0.33±0.04	0.46±0.04**	0.26±0.06	0.55±0.04**/▵
Rat 4	36	0.20±0.03	0.43±0.03**	0.26±0.04▵	0.51±0.04**/▵▵
Rat 5	28	0.30±0.07	0.55±0.07**	0.35±0.08	0.66±0.09**/▵
Rat 6	22	0.29±0.03	0.43±0.04*	0.24±0.07	0.54±0.07*

a The values are the mean ± SEM of the global efficiency values of each rats (paired sample *t* test).

b Total number of the compared groups.

* p<0.05 compared to the global efficiency values at the WMBS in the original dimensionality or the reduced dimensionality.

** p<0.01 compared to the global efficiency values at the WMBS in the original dimensionality or the reduced dimensionality.

▵ p<0.05 compared to the global efficiency values of the original dimensionality at the WMBS or at the WMS.

▵▵ p<0.01 compared to the global efficiency values of the original dimensionality at the WMBS or at the WMS.

**Table 3:** Comparisons of the causal density of original and reduced dimensionality at WMBS and WMS^a^.

	N^b^	Original dimensionality		Reduced dimensionality	
		WMBS	WMS	WMBS	WMS
Rat 1	26	0.24±0.02	0.35±0.02**	0.22±0.05	0.41±0.03**/▵
Rat 2	20	0.14±0.02	0.19±0.03	0.18±0.04	0.34±0.04**/▵▵
Rat 3	28	0.19±0.02	0.27±0.02**	0.17±0.04	0.40±0.04**/▵▵
Rat 4	36	0.16±0.02	0.25±0.02**	0.20±0.02▵	0.35±0.03**/▵▵
Rat 5	28	0.24±0.05	0.43±0.07*	0.27±0.06	0.55±0.08**/▵▵
Rat 6	22	0.18±0.01	0.23±0.02*	0.23±0.04	0.36±0.05*/▵

a The values are the mean ± SEM of the causal density values of each rats (paired sample *t* test).

b Total number of the compared groups.

* p<0.05 compared to the causal density values at the WMBS in the original dimensionality or the reduced dimensionality.

** p<0.01 compared to the causal density values at the WMBS in the original dimensionality or the reduced dimensionality.

▵ p<0.05 compared to the causal density values of the original dimensionality at the WMBS or at the WMS.

▵▵ p<0.01 compared to the causal density values of the original dimensionality at the WMBS or at the WMS.

Further investigations of the effectiveness by applying PCA showed that the values of the features were significant higher in the reduced dimensionality (E_PC_ and CD_PC_) at the WMS than they were in the original dimensionality (E and CD, Fig. 3C and D, 80 trials for 6 rats, paired sample t-test, ^**^ P<0.01). However, the differences between the features of the original and the reduced dimensionality were not statically significant at the WMBS (Fig. 3C and D, 80 trials for 6 rats, paired sample t-test, P>0.05). In addition, no significant differences were found between the features of the original and the reduced dimensionality at the WMBS except for rat 4 (Table 2 and Table 3, paired sample t-test, ^▵^P<0.05).

Thus, the average values of the features were both increased during the WM tasks. Besides, the variations of the functional network features were better described in the reduced dimensionality.

## Discussion

The results indicated increasing tendencies of the strength of the functional connectivity during the WM tasks. Besides, the functional connectivity (GC_PC_ and GC), effect of the parallel information transfer (E_PC_ and E) and global coordination (CD_PC_ and CD) had similar variation trends during the correct trials. The maximum values of these measures occurred at the same time (WMS). Therefore, stronger functional connectivity, higher information transfer efficiency and increased global coordination occurred at the WMS. In addition, significantly differences were found between the measure values of the WMS and the WMBS for all the rats in the reduced dimensionality. However, in the original space, statistical differences were only found in 5 rats, though the levels of the measures were higher at the WMBS for all the 6 rats. These results, taking together, suggested that combining the application of the PCA and the GCCA might provide an effective way to investigate the functional connectivity mechanism during the WM tasks in low dimensional space.

### Dynamic variations of functional connectivity during the incorrect WM tasks

To investigate the variations of the functional network during the incorrectly performed working memory tasks, we calculated the values of the features during the incorrect trials (20 trials for all 6 rats).

**Figure 4:**
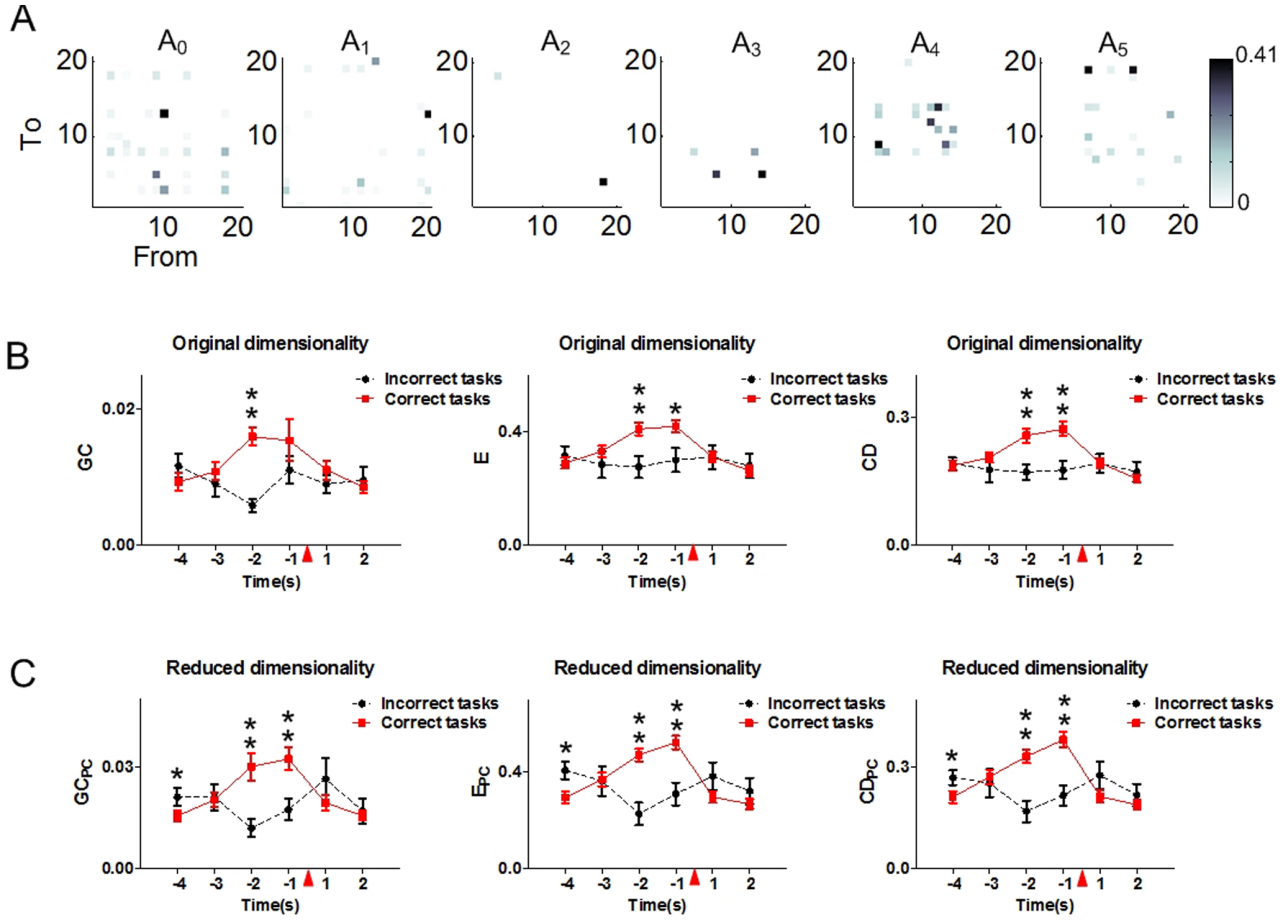
Dynamic variations of granger causality, global efficiency and causal density during the incorrect tasks in both the original and the reduced dimensionality (20 trials for 6 rats). The data are divided into six 1(4 s pre and 2 s post the tripping time). The red triangle indicates the tripping time of the infrared sensor in the Y-maze. (A) Dynamic variations of the granger causality matrixes during an incorrect trial of rat 1. (B) The variations of granger causality (left), global efficiency (middle) and causal density (right) during the incorrect tasks (20 trials for 6 rats) and the correct trials (80 trials for 6 rats) in the original dimensionality. The feature values in the correct trials are significantly higher 2 s (GC, E, CD) and 1 s (E, CD) pre the tripping time than those in the incorrect trials (t test, ^*^ P<0.05, ^**^ P<0.01). No statistical difference is found at the WMBS between the incorrect and the correct trials (t test, P>0.05). (C) The variations of granger causality (left), global efficiency (middle) and causal density (right) during the incorrect tasks (20 trials for 6 rats) and the correct trials (80 trials for 6 rats) in the reduced dimensionality. The feature values in the correct trials are significantly higher 2 s (GC_PC_, E_PC_, CD_PC_) and 1 s (GC_PC_, E_PC_, CD_PC_) pre the tripping time than those in the incorrect trials (t test, ^**^ P<0.01). In addition, the feature values were significantly higher at the WMBS in the incorrect trials (t test, ^*^ P<0.05).

As shown in Fig. 4A, the connections between the neurons varied during the incorrect working memory tasks. However, comparisons among different period feature values showed significant differences neither in the original dimensionality (one way ANOVA, GC: F_ (5,114)_ = 1.46, P = 0.21; E: F_ (5,114)_ = 0.16, P = 0.98; CD: F_ (5,114)_ = 0.14, P = 0.98) nor the reduced dimensionality (one way ANOVA, GC_PC_: F_ (5,114)_ = 1.54, P = 0.18; E_PC_: F_ (5,114)_ = 1.61, P = 0.16; CD_PC_: F_ (5,114)_ = 1.43, P = 0.22). Of note, in contrast with the variations of the network features in the correct trials, the feature values presented decreased tendencies during the incorrect trials. The feature levels of the correct (80 trials) and the incorrect (20 trials) working memory tasks were further compared. Results showed that there were no significant differences between the feature values at the WMBS in the original dimensionality (Fig. 4B, t test, P>0.05). However, in the reduced dimensionality, the feature values were much higher at the WMBS in the incorrect trials (Fig. 4C, t test, ^*^ P<0.05). Besides, the feature values were significantly higher in the correct trials 2 s or/and 1 s pre the tripping point both in the original and the reduced dimensionality (Fig. 4B and C, t test, ^*^ P<0.05, ^**^ P<0.01).

Therefore, during the error trials, no statistical differences were found between the network features of different task durations. Besides, the feature levels were much higher (1 s or 2 s pre the tripping point) in the correct trials.

Of note, Granger causality inferences are only valid if an MVAR model adequately captured the correlation structure in the data. The model could be considered as captured the data adequately if the sum-square-error was more than 0.3, the consistency value was above 80% and the value of Durbin -Watson statistic test was larger than 1.0, simultaneously [15]. In this study, these prerequisites had been satisfied.

In addition, the maximum values of the functional connectivity (GC), the effect of the parallel information transfer (E) and the global coordination (CD) appeared at A_3_ (Rat 1, Rat 4 and Rat 5) and A_2_ (Rat 2, Rat 3 and Rat 6), which corresponds to 2 s or 1 s before the tripping time. Because the tripping time of the infrared sensor marked the ‘choice run’ behavioral events during the WM tasks, the results suggested the strongest connectivity occurred at WMS happened 2 s or 1 s before the ‘choice’ behavioral events.

PCA is a powerful technique to extract components from all neurons based on the covariance structure. Investigations have demonstrated that the combination of the PCA and the GCCA might provide an effective way to study the brain effective connectivity [33]. Though the application of PCA will lead to a little loss of the information contained in original data, study has proved these will not affect the following connectivity analysis [34]. Besides, research suggests that the more efficient representation of the sequences generated by PCA indicates the more efficient information of relationship can be obtained in Granger causality analysis [24]. In the study, we use the PCA to extract the principle components in order to investigate whether the features of the functional connectivity during the WM tasks could be effectively described in a low dimension space. Results showed that the changing trends of GC_PC_, E_PC_ and CD_PC_ were the same as GC, E and CD (Fig. 2B, C, Fig. 3B and C). Besides, the maximum of GC_PC_, E_PC_ and CD_PC_ occurred at A_3_ (Rat 1, Rat 4 and Rat 5) or A_2_ (Rat 2, Rat 3 and Rat 6), which also just before the ‘choice’ behavioral events.

In order to detect the effectiveness of describing the functional connectivity by using PCA, the features obtained from the network constructed by the continuous spikes (GC, E and CD) and from the network constructed by the PCs (GC_PC_, E_PC_ and CD_PC_) were further compared, correspondingly. The values of GC_PC_ were significantly higher than the GC values at the WMBS (Fig. 2C). Meanwhile, no significant differences were found between the values of global efficiency (Fig. 3C) at the WMBS, as well as the causal density (Fig. 3D). Of note, additional investigations showed that statistical differences between the feature values of the original and the reduced dimensionality could only be found in some of the rats at the WMBS (Table 1, 2 and 3). By contrast, the features of the PCs constructed network were significantly lager than those of the continuous spikes constructed network at the WMS (Table 1, 2 and 3).

These findings suggested that PCA was an effective way to describe the features of the functional connectivity during WM, though the connections among the PCs were not identical to the underlying physical mechanism of the connections between the neurons. The approach of combining the PCA and the GCCA could be an effective way to investigate the mechanism of brain cognitive function by analyzing the features of the functional connectivity during WM.
